# Overlapping airway diseases and risk of major adverse cardiovascular events: a UK biobank prospective cohort study

**DOI:** 10.3389/fcvm.2026.1875022

**Published:** 2026-07-29

**Authors:** Pingsheng Hu, Linhua Kuang, Di-en Yan, Jianming Wu, Keyang Zheng, Junyu Pei, Wenqi He

**Affiliations:** 1 Department of Respiratory and Critical Care Medicine, Ji'an Central Hospital, Ji'an, China; 2 Department of Cardiology, Ji'an Central Hospital, Ji'an, China; 3 Department of Endocrinology, Ji'an Central Hospital, Ji'an, China; 4 Department of Intensive Care Unit, Ji'an Central Hospital, Ji'an, China; 5 Department of General Practice, Beijing Nuclear Industry Hospital, Beijing, China; 6 Department of Cardiovascular Medicine, The Second Xiangya Hospital, Central South University, Changsha, China; 7 Department of General Practice, Ji'an Central Hospital, Ji'an, China

**Keywords:** airway disease overlap, asthma, bronchiectasis, chronic obstructive pulmonary disease, major adverse cardiovascular events, prospective cohort study, sex differences, UK biobank

## Abstract

**Background:**

Asthma, chronic obstructive pulmonary disease (COPD), and bronchiectasis frequently coexist, but the cardiovascular implications of their overlap phenotypes have not been systematically quantified in large prospective cohorts.

**Methods:**

Among 407,127 UK Biobank participants free of cardiovascular disease at baseline, we classified asthma, COPD, and bronchiectasis into eight mutually exclusive phenotypes and used Cox proportional hazards regression to assess their associations with the first occurrence of major adverse cardiovascular events (MACE, comprising myocardial infarction, stroke, and cardiovascular death). We further tested for a dose–response relationship and effect modification by sex.

**Results:**

Over a median follow-up of 14 years, 19,660 MACE occurred. All overlap phenotypes carried elevated MACE risk, with asthma–bronchiectasis showing the highest risk (hazard ratio [HR] 3.23, 95% confidence interval [CI] 1.94–5.36); this association remained stable across sequential confounder adjustments, indicating that the risk was independent of traditional cardiovascular factors. MACE risk rose stepwise with the number of concurrent airway diseases (HRs of 1.00, 1.15, and 1.79 for 0, 1, and 2 diseases; *P*-trend = 1.45 × 10^−23^). Sex significantly modified this association (P-interaction = 0.0015), with consistently stronger risk elevations in women than in men across all principal phenotypes.

**Conclusions:**

Overlapping airway diseases substantially elevate MACE risk in a dose-dependent manner, with risk most pronounced in women. Asthma–bronchiectasis emerged as a previously under-recognised high-risk combination. These findings support integrating airway comorbidity into cardiovascular risk stratification.

## 1 Introduction

Cardiovascular disease (CVD) remains the leading cause of death globally, responsible for 19.2 million deaths in 2023, up from 13.1 million in 1990, with the global prevalent case count more than doubling over the same period (1). In parallel, chronic airway diseases—asthma, chronic obstructive pulmonary disease (COPD), and bronchiectasis—rank among the most prevalent non-communicable conditions worldwide, with asthma alone affecting an estimated 262 million individuals and COPD an additional 212 million globally, imposing a substantial and growing burden on global health (2, 3). These two disease domains intersect closely in clinical practice: epidemiological evidence indicates that patients with chronic airway disease are at markedly elevated risk of CVD, and CVD constitutes a leading cause of non-respiratory mortality in this population, pointing to shared pathophysiological underpinnings.

Asthma, COPD, and bronchiectasis differ in their inflammatory pathways and structural changes, and each shows a distinct pattern of association with CVD. Asthma is a heterogeneous disease characterised by variable respiratory symptoms and reversible expiratory airflow limitation, driven predominantly by type 2 (eosinophilic) inflammation. The resulting systemic inflammation has been linked epidemiologically to atherosclerotic events and adverse vascular outcomes (4–6). COPD, defined by persistent airflow limitation arising from chronic inflammatory abnormalities of the airways and alveoli (7), amplifies cardiovascular risk through multiple, partly inflammation-independent pathways—not only systemic inflammation and oxidative stress, but also hypoxaemia, hypercapnia, and lung hyperinflation, which impose haemodynamic load, sympathetic activation, and impaired ventricular filling. The rate of major adverse cardiovascular events (MACE) in patients with COPD is approximately 25% higher than in non-COPD individuals—comparable to the excess risk attributable to diabetes—and is further magnified during and in the months following acute exacerbations (7–9). Bronchiectasis is characterised by recurrent airway infection and irreversible bronchial dilation, framed by the “vicious vortex” model, in which infection, neutrophilic inflammation, impaired mucociliary clearance, and structural damage reciprocally reinforce one another (10). Bronchiectasis has also been linked to an elevated incidence of coronary heart disease and ischaemic stroke (11, 12).

These mechanistically distinct yet partly convergent inflammatory pathways—predominantly type 2 in asthma, and neutrophilic in both COPD and bronchiectasis but with differing upstream triggers (noxious inhalants in COPD, recurrent infection in bronchiectasis)—suggest that the coexistence of these conditions may produce a more severe systemic inflammatory state than any single disease alone, thereby amplifying cardiovascular risk. Indirect evidence supports this inference: asthma–COPD overlap has been associated with significantly higher risks of coronary heart disease and heart failure than either condition alone, as well as a disproportionate cardiovascular hospitalisation burden (13, 14). Whether this pattern generalises to the broader landscape of airway disease overlap, however, remains unclear. Whether cumulative airway disease burden is associated with MACE in a dose-dependent manner has not been systematically tested; whether overlap combinations involving bronchiectasis—particularly asthma–bronchiectasis—confer comparable independent risk has not been quantified (15); and given well-documented sex differences in both airway disease biology (16, 17) and cardiovascular risk (18, 19), whether sex modifies the airway disease–MACE relationship has yet to be examined in large prospective cohorts (20).

To address these gaps, we leveraged the UK Biobank prospective cohort (*N* = 407,127; median follow-up approximately 14 years) to systematically evaluate the associations of single and overlapping airway disease phenotypes with incident MACE, the dose–response relationship between cumulative airway disease burden and MACE risk, and the modifying role of sex.

## 2 Methods

### 2.1 Participants and study design

This was a prospective cohort analysis based on the UK Biobank, a large population-based cohort that recruited approximately 500,000 participants aged 40–69 years from 22 assessment centres across England, Scotland, and Wales between 2006 and 2010, with long-term follow-up enabled through linkage to National Health Service (NHS) records, including Hospital Episode Statistics (HES), primary care records, and death registries (21). The UK Biobank study was approved by the North West Multi-Centre Research Ethics Committee (reference 11/NW/0382), and all participants provided written informed consent at baseline. The present analysis was conducted under UK Biobank Application ID [1116314] and is reported in accordance with the Strengthening the Reporting of Observational Studies in Epidemiology (STROBE) guidelines.

From 501,936 participants assessed at baseline, we sequentially excluded individuals with prevalent cardiovascular disease at baseline (*n* = 39,173), those with missing baseline assessment date (*n* = 0), and those with complete missingness in key covariates that precluded imputation (*n* = 13,788), and those with missing values in covariates with <5% missingness handled by complete-case deletion (*n* = 41,848), leaving 407,127 participants for the primary analysis. Prevalent cardiovascular disease was defined as a diagnosis of myocardial infarction, stroke, coronary heart disease/angina, or heart failure at or before baseline, ascertained from the union of first-occurrence fields, self-reported non-cancer illness codes (field 20002), and inpatient ICD-10 records (field 41270); medication use was not used in the definition. Full code lists are provided in Supplementary Table S1.

### 2.2 Definition of airway diseases

Asthma, COPD, and bronchiectasis were each ascertained using a combination of self-report and ICD-10 codes, with positivity defined by either source. Self-reported diagnoses were derived from the baseline touchscreen questionnaire and verbal interview based on doctor-diagnosed conditions and self-reported non-cancer illnesses, while ICD-10–coded diagnoses were obtained through linkage to HES and primary care records. Specifically, asthma was defined by self-report (field 6,152; or non-cancer illness code 20,002 = 1,111) or ICD-10 codes J45/J46; COPD by self-report (20,002 = 1,112) or ICD-10 codes J41–J44; and bronchiectasis by self-report (20,002 = 1,144) or ICD-10 code J47. To preserve the temporal sequence of exposure ascertainment, only diagnoses recorded on or before the baseline assessment date were used to define baseline exposure, and airway diseases first diagnosed during follow-up were not classified as baseline exposure. Based on the presence or absence of the three diseases, participants were assigned to one of eight mutually exclusive groups: no airway disease, asthma alone, COPD alone, bronchiectasis alone, asthma–COPD overlap (ACO), asthma–bronchiectasis overlap, COPD–bronchiectasis overlap, and triple overlap.

### 2.3 Outcome ascertainment and follow-up

The primary outcome was the first occurrence of MACE, defined as the first event of myocardial infarction, stroke, or cardiovascular death. Outcomes were ascertained from linked HES inpatient records and the NHS Digital death registry, both based on objective clinical records and independent of self-report; participants with multiple MACE events during follow-up contributed only their first event to the analysis.

Each participant was followed from the baseline assessment date until the first occurrence of MACE, death from any cause, loss to follow-up, or the administrative censoring date, whichever came first. The HES administrative censoring date for the present analysis was 31 March 2023, yielding a median follow-up of approximately 14 years.

### 2.4 Data collection

Baseline information was collected uniformly at UK Biobank assessment centres between 2006 and 2010 through touchscreen questionnaires, verbal interviews, physical measurements, and biological sample collection. Demographic data included age, sex, and self-reported ethnicity (British, Other White, South Asian, Black, Mixed, or Other). Socioeconomic data included the Townsend deprivation index (derived from baseline postcode), educational attainment (degree or above, A-levels or vocational, General Certificate of Secondary Education [GCSE], or no qualification), and employment status. Lifestyle factors included smoking status (never, former, current), pack-years, passive smoking exposure, alcohol consumption frequency, physical activity (metabolic equivalent of task [MET]-min/week, derived from the short-form International Physical Activity Questionnaire [IPAQ]), self-reported sleep duration, and a 0–4 healthy diet score derived from the cumulative achievement of four dietary criteria covering vegetable, fruit, fish, and processed meat intake. Anthropometric measurements included height, weight, body mass index (BMI), and waist-to-hip ratio (WHR). Pulmonary function measurements included forced expiratory volume in 1 s (FEV1), forced vital capacity (FVC), and the FEV1/FVC ratio, performed using a Vitalograph Pneumotrac 6,800 spirometer in accordance with American Thoracic Society/European Respiratory Society (ATS/ERS) standards. Laboratory parameters included peripheral blood eosinophil count, C-reactive protein (CRP), fasting glucose, glycated haemoglobin (HbA1c), lipid profile (total cholesterol, low-density lipoprotein cholesterol [LDL-C], high-density lipoprotein cholesterol [HDL-C], and triglycerides), serum creatinine, and the estimated glomerular filtration rate (eGFR) calculated from creatinine using the Chronic Kidney Disease Epidemiology Collaboration (CKD-EPI) equation. Comorbidities and medical history were ascertained through integration of self-report, ICD-10 codes, and medication records, and included hypertension, diabetes, dyslipidaemia, chronic kidney disease (CKD), cancer, and depression.

### 2.5 Statistical analysis

The normality of continuous variables was assessed using the Shapiro–Wilk test. Normally distributed continuous variables were presented as mean ± standard deviation (SD) and compared using one-way analysis of variance (ANOVA), while skewed continuous variables were presented as median (Q1, Q3) and compared using the Kruskal–Wallis test; categorical variables were presented as numbers (percentages) and compared using the chi-squared test or Fisher's exact test. These baseline characteristics were summarised in the full cohort free of prevalent cardiovascular disease (*n* = 448,975), whereas all regression analyses described below were performed in the analytic sample after missing-data handling (*n* = 407,127).

The associations of airway disease groups with the first occurrence of MACE were evaluated using Cox proportional hazards regression, with the no–airway disease group as reference, and results were expressed as hazard ratios (HRs) with 95% confidence intervals (CIs). Three sequentially adjusted models were specified: Model 1 was adjusted for age, sex, and ethnicity; Model 2 was further adjusted for the Townsend deprivation index, educational attainment, smoking status, passive smoking, alcohol intake, BMI, physical activity, and diet score; Model 3 additionally adjusted for hypertension, diabetes, dyslipidaemia, CKD, and log-transformed CRP. The covariates included in Model 3 were selected based on clinical relevance and a significance level of *P* < 0.05 in univariate analyses. Overall model discrimination was assessed using Harrell's C-index, and the proportional hazards assumption was evaluated using a global test based on Schoenfeld residuals, which showed no material violation. We additionally examined the pattern of HR attenuation across the three sequentially adjusted models qualitatively, to provide context for interpreting the relative contribution of traditional risk factors to each phenotype's association with MACE.

To assess the dose–response relationship between cumulative airway disease burden and MACE risk, participants were re-categorised by the number of concurrent airway diseases (0, 1, 2, or 3), and this count was entered as an ordinal variable in the fully adjusted Cox model, with the linear trend tested through a P-trend statistic. Subgroup analyses were conducted within the three groups with adequate sample size (asthma alone, COPD alone, and ACO), stratified by age (< 60 vs. ≥60 years), sex (male vs. female), and smoking status (never vs. ever/current), with each subgroup model adjusting for all Model 3 covariates except the stratifying variable. Effect modification was formally tested by including a multiplicative exposure×stratifier interaction term in the full dataset, assessed using a likelihood ratio test, with the corresponding P-interaction reported. Missing covariate data were handled according to the proportion of missingness: covariates with <5% missingness were addressed by complete-case deletion, and covariates with 5%–25% missingness were imputed using multiple imputation by chained equations (MICE); no covariate exceeded 25%. The exposure (airway disease group) and outcome variables were fully observed and were entered as predictors in the imputation model but were not themselves imputed. Five imputed datasets were generated using the mice package; the analysis model was fitted within each dataset and the estimates were pooled using Rubin's rules. Variable-specific missing proportions are provided in Supplementary Table S2.

Statistical significance was defined as a two-tailed *P* < 0.05. All analyses were performed using R software (Version 4.3.1; The R Foundation; http://www.R-project.org), with primary use of the survival, mice, and rms packages.

## 3 Results

### 3.1 Study population and baseline characteristics

A total of 448,975 participants were included in the baseline characterisation, with a median follow-up of approximately 14 years. Participants were classified into eight mutually exclusive groups: no airway disease (*n* = 391,189, 87.1%), asthma alone (*n* = 50,339, 11.2%), COPD alone (*n* = 4,085, 0.91%), bronchiectasis alone (*n* = 145, 0.03%), ACO (*n* = 2,907, 0.65%), asthma-bronchiectasis (*n* = 134, 0.03%), COPD-bronchiectasis (*n* = 82, 0.02%), and triple overlap (*n* = 94, 0.02%). Median follow-up was balanced across groups (range: 13.49–14.05 years).

A total of 19,660 MACE events occurred. Crude event rates ranged from 4.27% in the reference group to 11.94% in the asthma-bronchiectasis group. Baseline characteristics are presented in Table 1. COPD-containing groups were older, had higher smoking burden, and greater comorbidity prevalence. CRP increased progressively from 1.27 mg/L in the reference group to 4.08 mg/L in the triple overlap group. FEV_1_ declined from 2.82 L to 1.80 L across groups. Bronchiectasis-containing groups had a higher proportion of females (64.6%–68.7%). All between-group comparisons were significant (all *P* < 0.001).

**Table 1:** Baseline characteristics of study participants by airway disease group (*N* = 448,975).

Variable	No Airway Disease	Asthma Alone	COPD Alone	Bronch. Alone	ACO	Asthma + Bronch.	COPD + Bronch.	Triple
N	391,189	50,339	4,085	145	2,907	134	82	94
MACE, *n* (%)	16,690 (4.3)	2,213 (4.4)	408 (10.0)	7 (4.8)	310 (10.7)	16 (11.9)	9 (11.0)	7 (7.4)
*Demographics*								
Age, years	56.16 ± 8.05	55.13 ± 8.24	60.25 ± 6.84	61.01 ± 6.36	59.49 ± 7.21	60.28 ± 6.01	60.71 ± 6.41	60.97 ± 5.97
Female, *n* (%)	218,183 (55.8)	29,911 (59.4)	1,984 (48.6)	97 (66.9)	1,662 (57.2)	92 (68.7)	53 (64.6)	56 (59.6)
British, *n* (%)	347,358 (88.8)	44,622 (88.6)	3,696 (90.5)	131 (90.3)	2,582 (88.8)	127 (94.8)	74 (90.2)	88 (93.6)
*Socioeconomic*								
Townsend Index	−2.23(−3.69–0.33)	−2.08(−3.64–0.62)	−0.77(−2.91–2.42)	−2.14(−3.56–0.96)	−0.65(−2.94–2.83)	−1.94(−3.52–0.20)	−2.23(−3.77–0.94)	−1.66(−3.64–2.70)
Degree, *n* (%)	131,751 (33.7)	18,042 (35.8)	705 (17.3)	41 (28.3)	527 (18.1)	46 (34.3)	14 (17.1)	12 (12.8)
No qualification (%)	61,892 (15.8)	7,461 (14.8)	1,488 (36.4)	42 (29.0)	1,011 (34.8)	27 (20.1)	25 (30.5)	36 (38.3)
Employed, *n* (%)	235,298 (60.1)	30,960 (61.5)	1,500 (36.7)	42 (29.0)	954 (32.8)	45 (33.6)	28 (34.1)	18 (19.1)
*Lifestyle*								
Smoking Status								
Never	2,20,329 (56.3%)	28,790 (57.2%)	963 (23.6%)	91 (62.8%)	867 (29.8%)	83 (61.9%)	48 (58.5%)	43 (45.7%)
Former	1,30,577 (33.4%)	17,138 (34.0%)	1,995 (48.8%)	44 (30.3%)	1,342 (46.2%)	44 (32.8%)	29 (35.4%)	41 (43.6%)
Current	40,283 (10.3%)	4,411 (8.8%)	1,127 (27.6%)	10 (6.9%)	698 (24.0%)	7 (5.2%)	5 (6.1%)	10 (10.6%)
Pack-years	0.00 (0.00–9.62)	0.00 (0.00–8.40)	25.00 (0.00–43.00)	0.00 (0.00–0.00)	19.50 (0.00–39.50)	0.00 (0.00–2.19)	0.00 (0.00–13.25)	0.00 (0.00–25.31)
BMI, kg/m²	27.17 ± 4.64	27.93 ± 5.21	27.60 ± 5.31	25.52 ± 4.99	28.81 ± 6.08	26.03 ± 4.54	26.14 ± 4.57	27.95 ± 5.80
WHR	0.87 ± 0.09	0.87 ± 0.09	0.90 ± 0.09	0.85 ± 0.09	0.91 ± 0.10	0.86 ± 0.09	0.87 ± 0.10	0.92 ± 0.11
Sleep, h	7.16 ± 1.07	7.10 ± 1.13	7.15 ± 1.40	7.19 ± 1.42	7.05 ± 1.56	7.32 ± 1.26	6.96 ± 1.32	7.26 ± 1.58
Diet score	1.66 ± 0.99	1.65 ± 0.98	1.49 ± 0.99	1.70 ± 0.96	1.55 ± 1.00	1.76 ± 1.01	1.71 ± 0.96	1.79 ± 0.98
*Laboratory*								
Eosinophil, ×10⁹/L	0.13 (0.10–0.20)	0.20 (0.10–0.30)	0.16 (0.10–0.24)	0.14 (0.10–0.22)	0.20 (0.10–0.30)	0.20 (0.10–0.34)	0.16 (0.10–0.26)	0.20 (0.10–0.32)
CRP, mg/L	1.27 (0.63–2.61)	1.51 (0.73–3.19)	2.17 (1.06–4.68)	2.96 (1.03–6.71)	2.73 (1.28–5.47)	2.86 (1.01–6.23)	3.17 (1.71–8.84)	4.08 (1.85–11.14)
Glucose, mmol/L	5.09 ± 1.16	5.09 ± 1.23	5.18 ± 1.42	5.00 ± 1.10	5.25 ± 1.45	5.06 ± 0.95	5.36 ± 1.99	5.39 ± 1.38
HbA1c, mmol/mol	35.76 ± 6.28	35.93 ± 6.47	37.53 ± 7.53	37.15 ± 6.31	38.00 ± 8.06	36.42 ± 4.75	38.14 ± 9.58	39.61 ± 8.96
TC, mmol/L	5.77 ± 1.11	5.76 ± 1.11	5.69 ± 1.15	5.59 ± 1.01	5.72 ± 1.16	6.03 ± 1.19	5.51 ± 1.18	5.73 ± 1.19
LDL-C, mmol/L	3.61 ± 0.85	3.60 ± 0.84	3.56 ± 0.89	3.44 ± 0.78	3.56 ± 0.89	3.73 ± 0.94	3.36 ± 0.90	3.49 ± 0.88
HDL-C, mmol/L	1.46 ± 0.38	1.47 ± 0.38	1.41 ± 0.39	1.52 ± 0.40	1.46 ± 0.40	1.63 ± 0.40	1.47 ± 0.37	1.55 ± 0.47
TG, mmol/L	1.47 (1.03–2.13)	1.48 (1.04–2.13)	1.64 (1.17–2.31)	1.28 (0.93–1.82)	1.60 (1.13–2.28)	1.25 (0.91–1.75)	1.24 (0.90–1.84)	1.53 (1.17–2.15)
eGFR, mL/min/1.73m²	95.13 ± 12.78	95.84 ± 12.91	92.37 ± 13.65	92.36 ± 13.81	92.64 ± 14.16	91.77 ± 12.77	90.36 ± 15.99	91.09 ± 17.33
*Lung Function*								
FEV1, L	2.82 ± 0.78	2.60 ± 0.78	2.25 ± 0.84	2.13 ± 0.75	2.04 ± 0.78	2.01 ± 0.79	1.85 ± 0.61	1.80 ± 0.62
FVC, L	3.70 ± 1.03	3.56 ± 1.07	3.27 ± 1.02	2.95 ± 0.95	3.05 ± 0.95	2.93 ± 1.00	2.73 ± 0.88	2.74 ± 0.84
FEV1/FVC	0.76 ± 0.07	0.73 ± 0.08	0.68 ± 0.12	0.72 ± 0.08	0.66 ± 0.12	0.69 ± 0.11	0.68 ± 0.10	0.66 ± 0.12
*Comorbidities*								
Hypertension (%)	93,609 (23.9)	12,825 (25.5)	1,355 (33.2)	44 (30.3)	1,086 (37.4)	41 (30.6)	27 (32.9)	38 (40.4)
Diabetes (%)	16,056 (4.1)	2,315 (4.6)	265 (6.5)	3 (2.1)	273 (9.4)	9 (6.7)	5 (6.1)	13 (13.8)
Dyslipidemia (%)	49,182 (12.6)	5,971 (11.9)	786 (19.2)	17 (11.7)	511 (17.6)	13 (9.7)	12 (14.6)	15 (16.0)
CKD (%)	4,773 (1.2)	589 (1.2)	101 (2.5)	5 (3.4)	75 (2.6)	2 (1.5)	3 (3.7)	5 (5.3)
Cancer (%)	31,675 (8.1)	4,033 (8.0)	458 (11.2)	24 (16.6)	381 (13.1)	23 (17.2)	14 (17.1)	10 (10.6)
Depression (%)	21,743 (5.6)	4,005 (8.0)	445 (10.9)	12 (8.3)	402 (13.8)	16 (11.9)	5 (6.1)	15 (16.0)

Continuous variables: mean ± SD or median (IQR). Categorical variables: *n* (%). All *P* < 0.001 across groups. ACO, asthma-COPD overlap; Bronch., bronchiectasis; BMI, body mass index; WHR, waist-to-hip ratio; CRP, C-reactive protein; TC, total cholesterol; TG, triglycerides; eGFR, estimated glomerular filtration rate; CKD, chronic kidney disease.

Presented for the full cohort without prevalent CVD (*N* = 448,975); the primary analysis included 407,127 participants after missing-data handling.

### 3.2 Association between airway disease patterns and MACE risk

**Figure 1:**
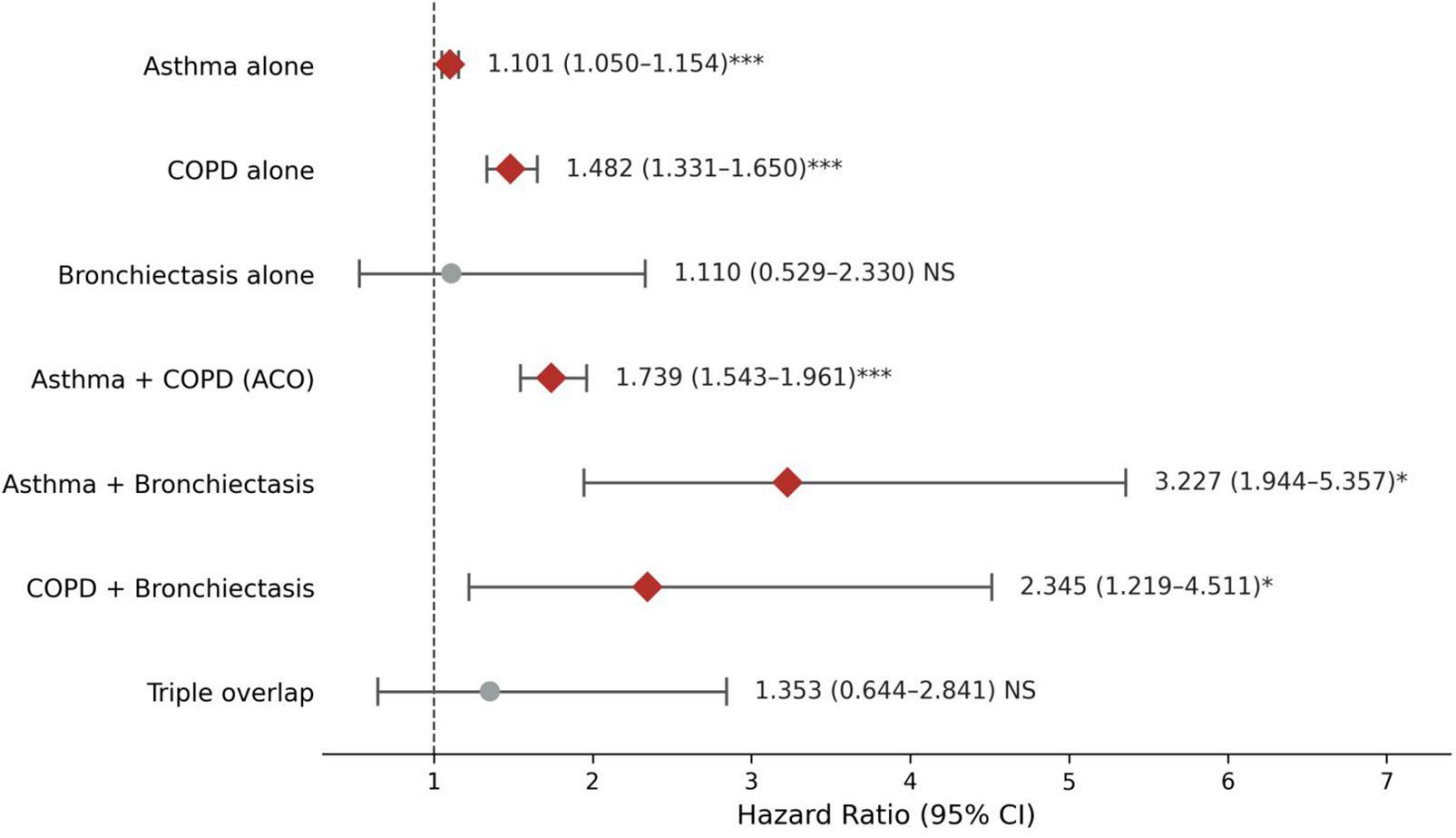
Fully adjusted hazard ratios (95% CI) for incident MACE by airway disease group (model 3). Red diamonds: statistically significant; gray circles: non-significant. Dashed line: HR = 1.0.

Results of the three sequentially adjusted Cox models are presented in Table 2 and Figure 1. In Model 3, COPD alone was associated with a HR of 1.482 (95% CI 1.331–1.650), and asthma alone with a HR of 1.101 (95% CI 1.050–1.154; both *P* < 0.001). Bronchiectasis alone was not significantly associated with MACE (HR 1.110, 95% CI 0.529–2.330).

**Table 2:** Hazard ratios (95% CI) for incident MACE across three Cox models.

Group	Model 1 HR (95% CI)	Model 2 HR (95% CI)	Model 3 HR (95% CI)
Asthma alone	1.128 (1.076–1.183)***	1.112 (1.061–1.166)***	1.101 (1.050–1.154)***
COPD alone	1.962 (1.764–2.183)***	1.530 (1.374–1.704)***	1.482 (1.331–1.650)***
Bronchiectasis alone	1.174 (0.560–2.464)	1.198 (0.571–2.514)	1.110 (0.529–2.330)
ACO	2.324 (2.063–2.619)***	1.828 (1.621–2.061)***	1.739 (1.543–1.961)***
Asthma + Bronch.	3.170 (1.911–5.260)*	3.541 (2.133–5.877)*	3.227 (1.944–5.357)*
COPD + Bronch.	2.773 (1.443–5.332)**	2.728 (1.418–5.245)**	2.345 (1.219–4.511)*
Triple overlap	1.924 (0.917–4.036)	1.625 (0.774–3.411)	1.353 (0.644–2.841)
C-index	0.700	0.725	0.733

Reference: no airway disease. Model 1: age, sex, ethnicity. Model 2: + Townsend, education, smoking, passive smoking, alcohol, BMI, physical activity, diet score. Model 3: + hypertension, diabetes, dyslipidaemia, CKD, CRP.

* *P* < 0.05.

** *P* < 0.01.

*** *P* < 0.001.

Among overlap groups, most demonstrated significantly elevated MACE risk, with the exception of the triple overlap group (HR 1.353, 95% CI 0.644–2.841). The asthma-bronchiectasis combination had the highest HR (3.227, 95% CI 1.944–5.357, *P* < 0.05), with HRs that remained consistently above 3 across all three models (3.170, 3.541, and 3.227). ACO had a HR of 1.739 (95% CI 1.543–1.961, *P* < 0.001), and COPD-bronchiectasis a HR of 2.345 (95% CI 1.219–4.511, *P* < 0.05).

**Figure 2:**
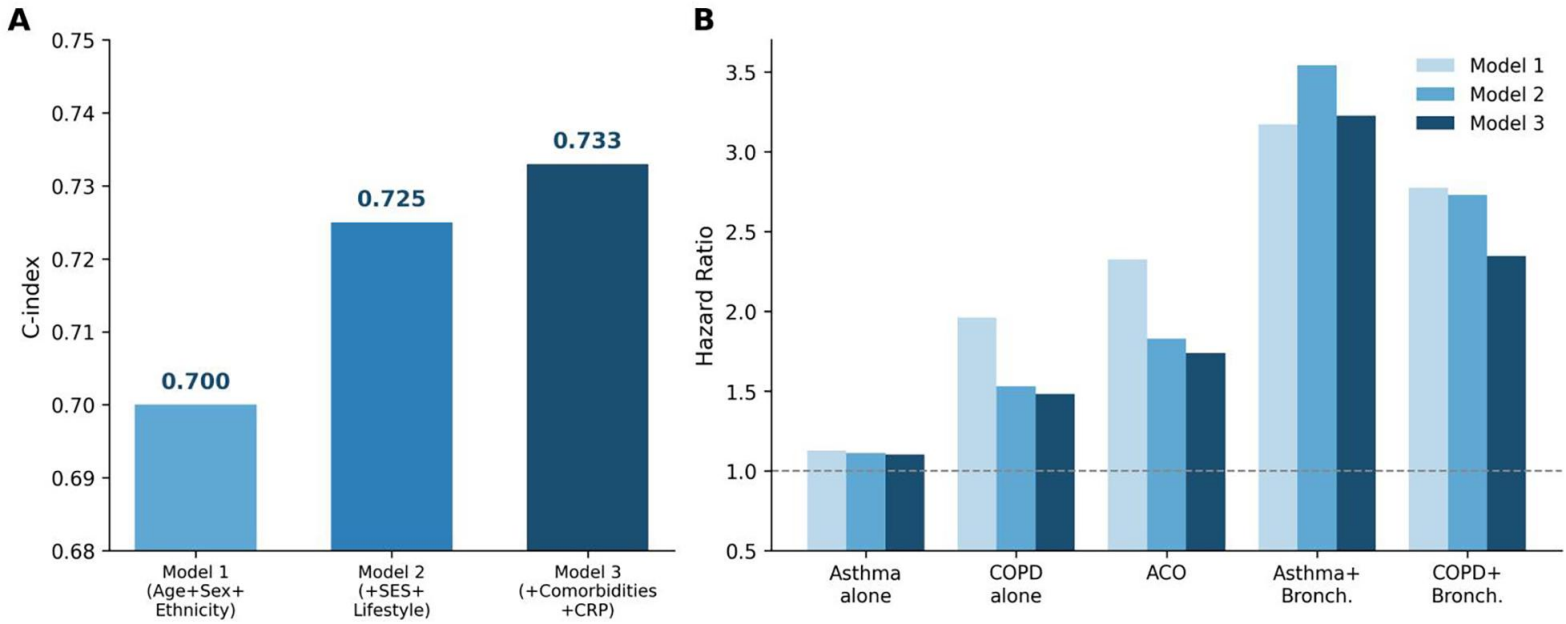
**(A)** Harrell's C-index across three models. **(B)** HR attenuation from Model 1 to Model 3 for the five statistically significant groups.

The C-index improved from 0.700 (Model 1) to 0.725 (Model 2) and 0.733 (Model 3). HR attenuation from Model 1 to Model 3 was most pronounced for COPD alone (1.962 → 1.482) and ACO (2.324 → 1.739), whereas the asthma-bronchiectasis HR remained consistently elevated across all models (Figure 2).

### 3.3 Dose-Response relationship

**Figure 3:**
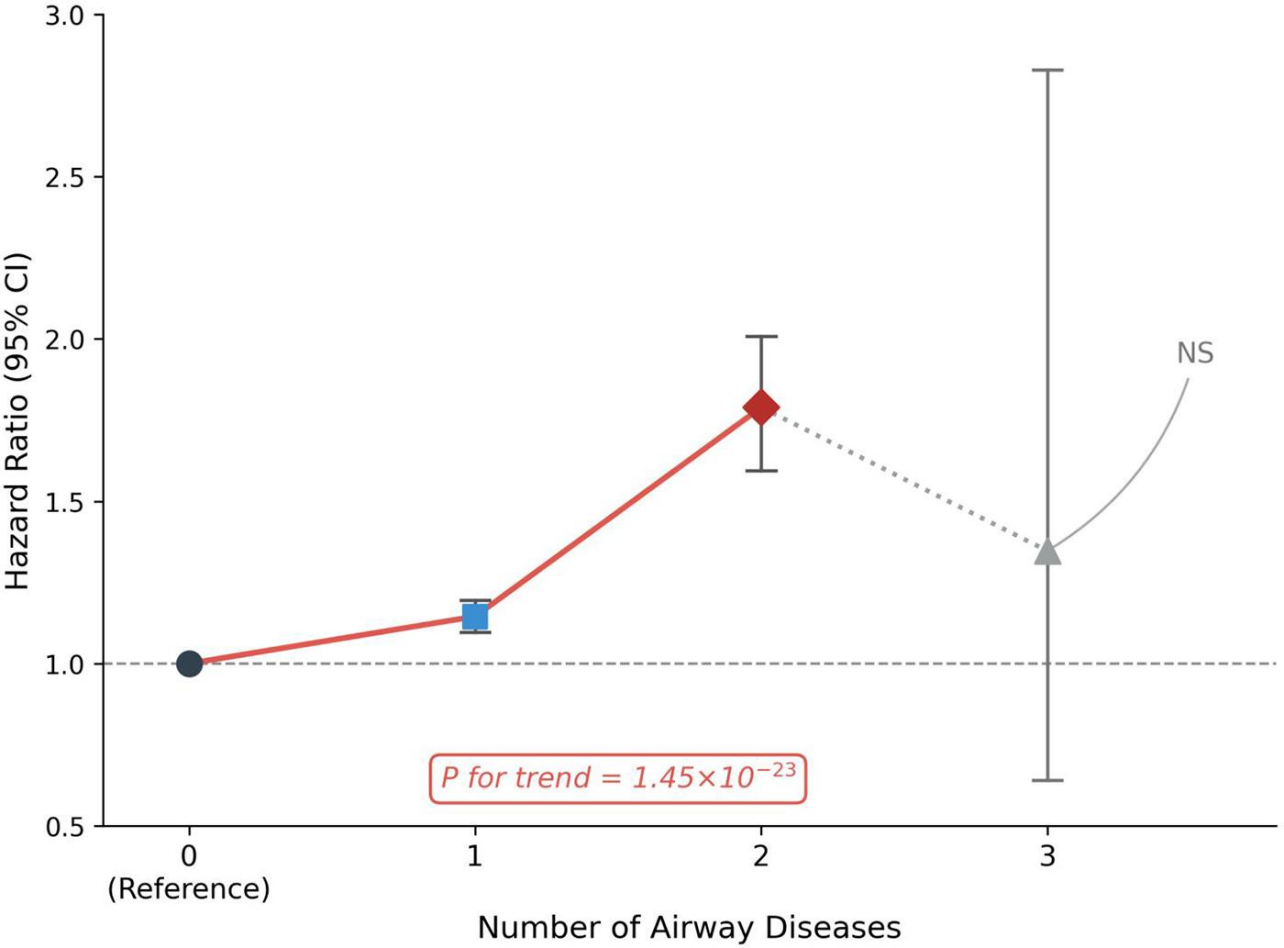
Dose-response relationship between the number of concurrent airway diseases and MACE risk (model 3). Solid line connects groups 0–2 (significant trend). Dotted line extends to group 3 (non-significant). *P* for trend = 1.45 × 10^−23^.

The HR increased stepwise with the number of concurrent airway diseases: 1 disease, HR 1.145 (95% CI 1.096–1.196); 2 diseases, HR 1.789 (95% CI 1.594–2.008; both *P* < 0.001). The *P* for trend was 1.45 × 10^−23^. The three-disease group did not reach significance (HR 1.347, 95% CI 0.641–2.829, *P* = 0.431), likely due to limited statistical power (Table 3, Figure 3).

**Table 3:** Dose-Response analysis: number of concurrent airway diseases and MACE risk.

Number of Diseases	HR (95% CI)	*P* value
0 (Reference)	1.00 (Reference)	—
1	1.145 (1.096–1.196)	<0.001
2	1.789 (1.594–2.008)	<0.001
3	1.347 (0.641–2.829)	0.431
**P for trend**	**1.45** **×** **10^−23^**	

Adjusted for Model 3 covariates. 0 = no airway disease; 1 = any single disease; 2 = any two-disease combination; 3 = triple overlap.

### 3.4 Subgroup analyses

**Figure 4:**
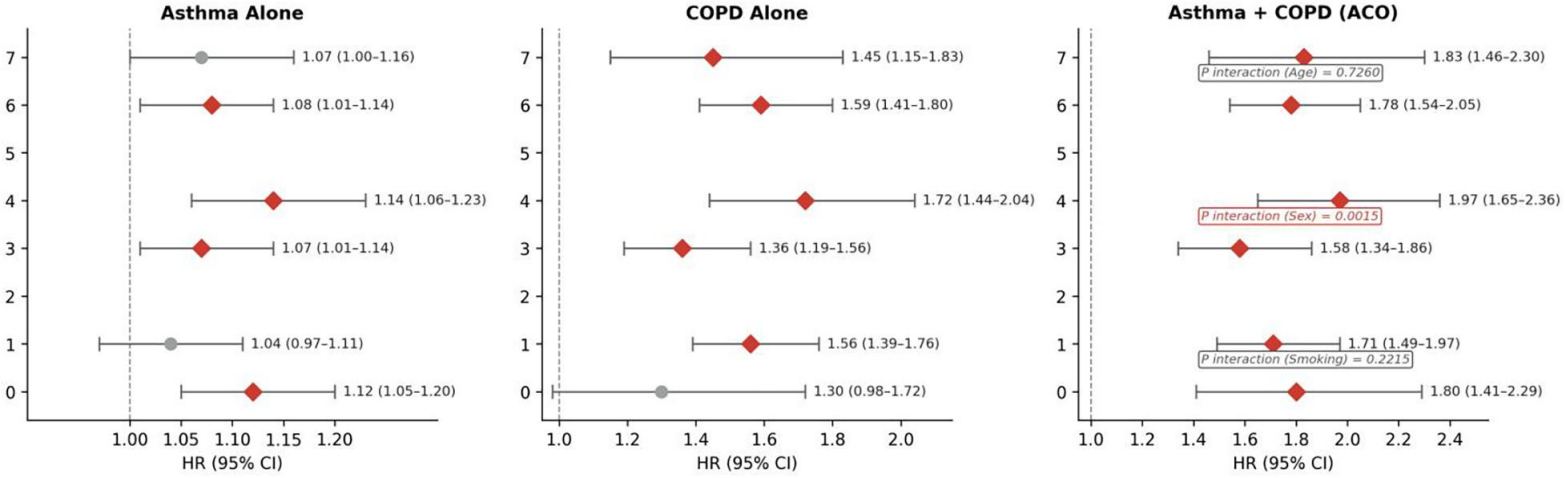
Subgroup analyses for asthma alone, COPD alone, and ACO stratified by age, sex, and smoking status. Red diamonds: significant; gray circles: non-significant.

Subgroup analyses for asthma alone, COPD alone, and ACO are presented in Table 4 and Figure 4. Sex was a significant effect modifier (*P* for interaction = 0.0015), with females consistently showing higher HRs than males across all three groups: asthma alone (1.14 vs. 1.07), COPD alone (1.71 vs. 1.36), and ACO (1.97 vs. 1.58).

**Table 4:** Subgroup analyses: HRs for MACE stratified by age, sex, and smoking status.

Subgroup	Level	*P* interaction	Asthma Alone HR (95% CI)	COPD Alone HR (95% CI)	ACO HR (95% CI)
Age	<60		1.07 (1.00–1.16)	1.45 (1.15–1.83)**	1.83 (1.46–2.30)***
	≥60	0.726	1.08 (1.01–1.14)*	1.59 (1.41–1.80)***	1.78 (1.54–2.05)***
Sex	Female		1.14 (1.06–1.23)***	1.71 (1.44–2.04)***	1.97 (1.65–2.36)***
	Male	0.002	1.07 (1.01–1.14)*	1.36 (1.19–1.56)***	1.58 (1.34–1.86)***
Smoking	Ever/Current		1.04 (0.97–1.11)	1.56 (1.39–1.76)***	1.71 (1.49–1.97)***
	Never	0.222	1.13 (1.05–1.21)***	1.30 (0.98–1.72)	1.80 (1.41–2.29)***

Reference: no airway disease within same stratum. Adjusted for Model 3 covariates except the stratification variable. *P* for interaction tested jointly across all three exposure groups. *P* for interaction values are displayed in the row of the second stratum level and apply to the entire subgroup variable.

* *P* < 0.05.

** *P* < 0.01.

*** *P* < 0.001.

No significant effect modification was observed for age (*P* = 0.726) or smoking status (*P* = 0.222). Of note, asthma alone did not reach significance in participants younger than 60 years (HR 1.07, 95% CI 1.00–1.16, *P* = 0.057). Among never-smokers, COPD alone was also non-significant (HR 1.30, 95% CI 0.98–1.72, *P* = 0.070).

## 4 Discussion

In this prospective cohort study of 407,127 UK Biobank participants with a median follow-up of approximately 14 years, we systematically evaluated the associations between single and overlapping phenotypes of three airway diseases—asthma, COPD, and bronchiectasis—and incident MACE. All overlap phenotypes carried elevated MACE risk relative to participants without airway disease, with the asthma–bronchiectasis combination conferring the highest risk (fully adjusted HR = 3.23, 95% CI 1.94–5.36), an association that remained stable across sequential confounder adjustments. Cumulative airway disease burden showed a strong dose–response relationship with MACE risk (P-trend = 1.45 × 10^−23^), and the association was consistently stronger in women than in men (P-interaction = 0.0015), whereas no meaningful effect modification was observed for age or smoking status.

The associations we observed for single airway diseases (asthma HR = 1.10; COPD HR = 1.48; bronchiectasis HR = 1.11) are broadly consistent with prior large cohort studies (8, 12, 22). For asthma–COPD overlap (ACO), our HR of 1.74 aligns with previous reports showing higher risks of coronary heart disease, heart failure, and cardiovascular hospitalisation in ACO compared with either condition alone (13, 14); in a nationwide cohort of 52,386 patients, Bonnesen et al. further reported that asthma as a comorbidity in severe COPD increased the risk of major cardiovascular events by 20%–25% (23). Prior ACO–CVD studies have largely relied on cross-sectional data or administrative databases; in contrast, the present study applied three sequentially adjusted Cox models in a large prospective cohort, allowing more rigorous control of multilevel confounding.

Overlap combinations involving bronchiectasis represent the most important extension of this work. Prior research on bronchiectasis–COPD overlap has focused predominantly on exacerbation frequency, hospitalisation, and all-cause mortality rather than cardiovascular-specific outcomes—within the European Multicentre Bronchiectasis Audit and Research Collaboration (EMBARC) registry of 16,730 patients with bronchiectasis, those with concomitant COPD experienced markedly higher risks of exacerbation and hospitalisation (24), and population-based studies of bronchiectasis–COPD overlap (BCOS) have reported elevated risks of heart disease and stroke (25, 26). Evidence on asthma–bronchiectasis overlap is even more limited: a Korean nationwide cohort and a meta-analysis have shown higher exacerbation risk and disease severity than asthma alone (27, 28), yet systematic evaluation of cardiovascular outcomes in large prospective cohorts has been lacking; a smaller bronchiectasis cohort (*n* = 250) reported a 29.6% rate of cardiovascular events during and after exacerbations, suggesting a tight link between acute airway inflammation and cardiovascular events (29). The present study is the first to quantify, in a large prospective cohort, the MACE risk associated with all overlap combinations involving bronchiectasis, and addresses key evidence gaps regarding dose–response and sex-based effect modification.

We observed that MACE risk rose stepwise with the number of concurrent airway diseases (HR 1.00 → 1.15 → 1.79 for 0, 1, and 2 diseases; P-trend = 1.45 × 10^−23^), indicating that the cumulative burden of airway disease itself constitutes an independent dimension of cardiovascular risk. In parallel, baseline median C-reactive protein (CRP) increased monotonically from 1.27 mg/L in the reference group to 4.08 mg/L in the triple-overlap group, while FEV1 declined monotonically from 2.82 L to 1.80 L, producing a dual gradient of intensifying systemic inflammation and worsening lung function. This pattern fits two well-established pathways through which cumulative airway disease may amplify cardiovascular risk: chronic inflammation, captured by CRP, accelerates atherogenesis through endothelial dysfunction, plaque instability, and a prothrombotic state (30, 31), while impaired lung function—itself an independent predictor of cardiovascular events—superimposes haemodynamic and oxygenation-related injury (32, 33). Against this background, the triple-overlap group did not follow the gradient, likely reflecting several converging factors. Beyond its small size and low event count, these patients carried the heaviest disease burden and therefore a higher competing risk of non-cardiovascular death; such patients may die from, or be censored for, non-cardiovascular causes before a MACE event occurs, attenuating the observed MACE hazard. Notably, the monotonic CRP and FEV1 trends described above persisted into this group, indicating that the non-significant hazard reflects limited statistical power and competing risk rather than a true reversal of the underlying biological gradient. Clinically, this dose–response relationship offers a risk-stratification signal that can be applied directly in routine respiratory practice: the number of coexisting airway diseases is itself a simple screening indicator of cardiovascular risk, complementary to conventional risk-factor scores.

Among the eight groups, asthma–bronchiectasis overlap carried the highest MACE risk (Model 3 HR = 3.23, 95% CI 1.94–5.36), with HRs essentially stable across the three sequential models (3.17, 3.54, and 3.23). In sharp contrast, the HRs for COPD-alone (1.96 → 1.48) and ACO (2.32 → 1.74) attenuated markedly after multivariable adjustment. The attenuation in COPD-alone and ACO suggests that much of their excess cardiovascular risk is mediated by traditional risk factors (smoking, hypertension, diabetes, socioeconomic deprivation), whereas the persistence of an HR above 3 for asthma–bronchiectasis suggests its risk reflects distinct, airway-disease-specific pathways. A plausible mechanism is the coexistence of “dual-axis inflammation”: the type 2/eosinophilic axis of asthma combined with the neutrophilic and chronic-infection axis of bronchiectasis may generate a composite systemic inflammatory burden exceeding either axis alone, driving cardiovascular injury through direct endothelial damage, accelerated atherosclerosis, and a prothrombotic milieu. This interpretation is consistent with recent reviews and cohorts of asthma–bronchiectasis overlap (27, 28, 34), which describe a more severe disease course, more frequent exacerbations, accelerated lung-function decline, and dual-axis inflammation with microbiome dysbiosis. Clinically, cardiovascular risk in asthma–bronchiectasis patients may be systematically underestimated by traditional risk-factor-based scores such as SCORE2 or the Pooled Cohort Equations.

Within the three principal disease categories—asthma, COPD, and ACO—women consistently showed higher MACE risks than men (asthma: 1.14 vs. 1.07; COPD: 1.71 vs. 1.36; ACO: 1.97 vs. 1.58; P-interaction = 0.0015). This sex difference is consistent with prior evidence: in the Framingham Offspring cohort, the asthma–CVD association reached significance only in women (HR 1.37 in women vs. 1.22 in men) (35), and a meta-analysis of 30 cohort studies found a 39% CVD risk increase among women with asthma vs. 19% in men (36). Our study formally confirms this sex difference in a large prospective cohort and is the first to extend it to COPD and ACO. Several mechanisms may contribute (37): women have intrinsically narrower airways and a biphasic response to oestrogen that may aggravate inflammation after menopause, and coronary heart disease in women more commonly involves microvascular disease and ischaemia with non-obstructive coronary arteries (INOCA), conditions closely linked to systemic inflammation but less reliably detected by conventional coronary assessment. Our findings indicate that women with airway disease—particularly those who are postmenopausal or carry overlap phenotypes—warrant more proactive cardiovascular evaluation.

Taken together, these findings carry implications at three levels. At the individual level, respiratory clinics should integrate cardiovascular risk assessment into the routine care of patients with airway disease, with particular attention to those with overlap phenotypes and to women. At the guideline level, the most recent GOLD report has incorporated cardiovascular assessment into the standard management of COPD; our dose–response evidence and the high-risk signal in asthma–bronchiectasis support extending this approach to the cardiovascular comorbidity sections of the Global Initiative for Asthma (GINA) and European Respiratory Society (ERS) bronchiectasis guidelines, which currently address this domain only sparingly. At the paradigm level, airway disease should not be viewed as a respiratory issue alone but as an indicator phenotype of systemic inflammatory burden, to be integrated into broader health risk assessment frameworks and to support a clinical shift from single-disease management toward multisystem comorbidity care (38).

The principal strengths of this study include its large prospective design (*N* = 407,127) with median follow-up of approximately 14 years, the systematic evaluation of all eight single and overlap combinations of the three airway diseases, the use of three sequentially adjusted Cox models to disentangle the contribution of traditional risk factors, and the objective ascertainment of MACE through HES and death registry linkage. Several limitations should nevertheless be considered. First, the smaller sample sizes in rare combinations (bronchiectasis-only, COPD–bronchiectasis, and triple overlap) limited statistical power; the non-significant HR in the triple-overlap group (HR = 1.35, *P* = 0.43) likely reflects limited statistical power and a higher competing risk of non-cardiovascular death rather than a true null effect, an interpretation supported by the strong dose–response trend. Although the asthma–bronchiectasis subgroup was modest in size, the consistency of HRs across three independently adjusted models argues against a chance finding. Second, UK Biobank is subject to a healthy-participant bias that may underestimate absolute risk, although prior work has shown that relative-risk estimates from UK Biobank remain robust compared with more representative population cohorts (39). Third, airway disease exposures were defined at baseline and do not capture changes during follow-up; such misclassification typically biases estimates toward the null, suggesting our HRs are conservative. Fourth, we lacked longitudinal data on pathway-specific inflammatory markers (e.g., interleukin [IL]-5, IL-6, IL-13), so the inferred “dual-axis inflammation” mechanism rests on biological plausibility rather than direct molecular evidence. In addition, our exposures were defined from self-reported and ICD-coded diagnoses without medication- or duration-based criteria, and prevalent cardiovascular disease was excluded by diagnostic codes without separately identifying participants who had undergone revascularisation (PCI or CABG) without a recorded MI or stroke; both choices follow prior UK Biobank studies and would bias estimates toward the null. Finally, more than 88% of UK Biobank participants are of European ancestry, and extrapolation to other ethnic groups should be made with caution.

In summary, in this large prospective UK Biobank cohort, overlapping airway diseases were associated with substantially elevated MACE risk in a strong dose–response manner, with consistently stronger associations among women. Asthma–bronchiectasis emerged as a previously under-recognised high-risk combination, with risk that was independent of traditional cardiovascular factors and suggestive of distinct, airway-disease-specific pathways. These findings support the integration of airway comorbidity into cardiovascular risk stratification and the broader clinical shift from single-disease management toward multisystem comorbidity care.

## Data Availability

Publicly available datasets were analyzed in this study. This data can be found here: The dataset analyzed for this study can be found in the UK Biobank (https://www.ukbiobank.ac.uk/). Access to the database was granted upon reasonable application and approval (application number: 1116314).
