# Supplementary material for: Overlapping airway diseases and risk of major adverse cardiovascular events: a UK biobank prospective cohort study
Source: Front Cardiovasc Med. 2026 Jul 29;13:1875022. doi: 10.3389/fcvm.2026.1875022 (PMC13461461; doi:10.3389/fcvm.2026.1875022)
Supplement: Supplementary file 1 [file Datasheet1.docx]

Supplementary Material

# 1 Supplementary Figures and Tables

## 1.1 Supplementary Tables

**Supplementary Table S1.** Code lists used to define airway disease exposures and to identify prevalent cardiovascular disease excluded at baseline, UK Biobank.

| **Condition** | **Self-report code** | **ICD-10 code** | **Data source field(s)** |
| --- | --- | --- | --- |
| **Part A. Airway disease exposures** | | | |
| Asthma | 1111 | J45, J46 | 6152; 20002; 41270 |
| COPD | 1112 | J41–J44 | 20002; 41270 |
| Bronchiectasis | 1144 | J47 | 20002; 41270 |
| **Part B. Prevalent cardiovascular disease (baseline exclusion)** | | | |
| Myocardial infarction | 1075 | I21, I22, I23 | 131298; 20002; 41270 |
| Stroke | 1583, 1483 (G45, TIA) | I60–I64 | 131368; 20002; 41270 |
| Coronary heart disease / angina | 1066, 1067, 1074 | I20, I24, I25 | 20002; 41270 |
| Heart failure | 1076 | I50 | 20002; 41270 |
| Other vascular disease | 1082 (PVD), 1083 (claudication) | I48 (atrial fibrillation) | 20002; 41270 |

**Notes:** Each condition was defined as present if identified by any of the listed sources (self-reported diagnosis or hospital ICD-10 record). Self-report codes are UK Biobank non-cancer illness codes (field 20002); for asthma, self-report could also be captured through field 6152. ICD-10 diagnoses were obtained from hospital inpatient records (field 41270); fields 131298 and 131368 denote the algorithmically derived first-occurrence dates for myocardial infarction and stroke, respectively. No duration- or medication-based criteria were applied to define any condition.

Prevalent cardiovascular disease was defined as the union of the three sources shown (first-occurrence field, self-report, and inpatient ICD-10); medication use was not used in the definition. Participants with any of the conditions listed in Part B at or before baseline were excluded from the analysis.

COPD, chronic obstructive pulmonary disease; ICD-10, International Classification of Diseases, 10th Revision; PVD, peripheral vascular disease; TIA, transient ischaemic attack.

**Supplementary Table S2.** Missing data proportions for baseline covariates and the handling strategy applied, UK Biobank.

| **Variable** | **Missing (%)** | **Handling** |
| --- | --- | --- |
| **Covariates with 5–25% missingness — multiple imputation (MICE)** | | |
| Physical activity | 21.7 | Imputed |
| Pack-years | 14.8 | Imputed |
| Fasting glucose | 13.8 | Imputed |
| HDL cholesterol | 13.7 | Imputed |
| Household income | 13.4 | Imputed |
| Passive smoking | 9.7 | Imputed |
| FVC | 8.2 | Imputed |
| FEV1 | 8.2 | Imputed |
| FEV1/FVC ratio | 8.2 | Imputed |
| HbA1c | 6.3 | Imputed |
| C-reactive protein | 5.9 | Imputed |
| LDL cholesterol | 5.9 | Imputed |
| Education | 5.8 | Imputed |
| Triglycerides | 5.8 | Imputed |
| Creatinine | 5.8 | Imputed |
| eGFR | 5.8 | Imputed |
| Cystatin C | 5.7 | Imputed |
| Total cholesterol | 5.7 | Imputed |
| Systolic blood pressure | 5.6 | Imputed |
| Diastolic blood pressure | 5.6 | Imputed |
| **Covariates with <5% missingness — complete-case deletion** | | |
| Eosinophil count | 4.2 | Row deleted |
| Employment status | 2.9 | Row deleted |
| Vegetable score | 1.5 | Row deleted |
| Sleep duration | 0.5 | Row deleted |
| BMI | 0.5 | Row deleted |
| Waist-to-hip ratio | 0.3 | Row deleted |
| Hip circumference | 0.3 | Row deleted |
| Waist circumference | 0.3 | Row deleted |
| Processed meat score | 0.2 | Row deleted |
| Fruit score | 0.2 | Row deleted |
| Fish score | 0.2 | Row deleted |
| Alcohol intake | 0.1 | Row deleted |
| Diet score | 0.01 | Row deleted |
| **Exposure and outcome variables — complete (no missing data)** | | |
| Airway disease group (exposure) | 0 | — |
| MACE event and follow-up time (outcome) | 0 | — |

**Notes:** Missingness was handled according to its proportion: covariates with 5–25% missingness were imputed using multiple imputation by chained equations (MICE); covariates with <5% missingness were addressed by complete-case deletion of the affected rows; no covariate exceeded 25% missingness. The exposure (airway disease group) and outcome variables (MACE event and follow-up time) were fully observed and were included as predictors in the imputation model but were not themselves imputed. Five imputed datasets were generated; the analysis model was fitted within each dataset and estimates were pooled using Rubin's rules.

BMI, body mass index; eGFR, estimated glomerular filtration rate; FEV1, forced expiratory volume in 1 second; FVC, forced vital capacity; HbA1c, glycated haemoglobin; HDL, high-density lipoprotein; LDL, low-density lipoprotein; MACE, major adverse cardiovascular event; MICE, multiple imputation by chained equations.
